# Suppression of P-cadherin expression as a key regulatory element for embryonic stem cell stemness

**DOI:** 10.1247/csf.22060

**Published:** 2022-12-28

**Authors:** Yuka Takeda, Shuji Matsuguchi, Sae Nozaki, Taisei Mihara, Junya Abe, Yohei Hirai

**Affiliations:** 1 Department of Biomedical Sciences, Graduate School of Science and Technology, Kwansei Gakuin University, 1 Gakuen Uegahara, Sanda, 669-1330, Japan

**Keywords:** differentiation, embryoid body, ES cells, P-cadherin, syntaxin4

## Abstract

In embryonic stem (ES) cell colonies, a small subpopulation that changes cell shape and loses pluripotency often appears in two-dimensional (2D) cultures, even in the presence of a stemness factor. We have previously shown that membrane translocation of the syntaxin4, t-SNARE protein contributes to this phenomenon. Here, we show that ES cells in three-dimensional (3D) aggregates do not succumb to extruded syntaxin4 owing to suppressed expression of P-cadherin protein. While extracellular expression of syntaxin4 led to the striking upregulation of P-cadherin mRNA in both 2D and 3D-ES cells, morphological changes and appreciable expression of P-cadherin protein were detected only in 2D-ES cells. Importantly, the introduction of an expression cassette for P-cadherin practically reproduced the effects induced by extracellular syntaxin4, where the transgene product was clearly detected in 2D-, but not 3D-ES cells. An expression construct for P-cadherin-Venus harboring an in-frame insertion of the P2A sequence at the joint region gave fluorescent signals only in the cytoplasm of 2D-ES cells, demonstrating translational regulation of P-cadherin. These results provide the mechanistic insight into the uncontrollable differentiation in 2D-ES cells and shed light on the validity of the “embryoid body protocol commonly used for ES cell handling” for directional differentiation.

## Introduction

Pluripotent embryonic stem (ES) cells are established from the three-dimensional (3D) inner cell masses (ICM) of blastocysts, an early stage of mammalian embryos. These cells are classified into two groups. Naïve ES cells from pre-implantation embryos have great differentiation potential. Prime state ES cells are from the post-implantation epiblast (Kinoshita and Smith, 2018). In 3D-cell aggregates, called embryoid bodies (EBs), the entire cell population participates in cellular arrangement to respond to differentiation stimuli for lineage specification and segregation into three germ cell layers (Brickman and Serup, 2017; Karanfil and Bagci-Onder, 2016). In contrast, when these cells are cultured in two-dimensional (2D) culture dishes, a small cell subpopulation that changes the morphology and undergoes heterogeneous differentiation often appears spontaneously. This differentiation occurs even if the medium contains specific stemness factors, such as feeder cells, leukemia inhibitory factor (LIF), or inhibitors of GSK3-β/MEK1/2 (2i) for naïve state-ES cells, and FGF and activin for prime state-ES cells (Gafni *et al.*, 2013; Martello and Smith, 2014). We have previously found that syntaxin4, a plasmalemmal t-SNARE protein, is abundantly expressed in naïve ES cells, locally and temporally translocated across the cell membrane and triggers cell dissociation with loss of pluripotency. We also found that these cells decreased E-cadherin expression and are prone to differentiation into mesodermal lineages. Transcriptome analyses revealed that extracellular extrusion of syntaxin4 occurs concomitantly with steep upregulation of P-cadherin, a classical cadherin (Hagiwara-Chatani *et al.*, 2017). During early embryogenesis, the expression of P-cadherin protein reportedly substitutes for that of E-cadherin around the primitive streak upon gastrulation and becomes detectable in emerging mesoderm (Acloque *et al.*, 2017; Moly *et al.*, 2016). Together with the fact that ES cells are equivalent to cells in ICM or epiblast and that syntaxin4-deficiency results in an embryonic lethal at the onset of gastrulation (Yang *et al.*, 2001), it is quite possible that an extrusion of syntaxin4 followed by P-cadherin expression is closely related to this event.

While either naïve or prime state ES cells express E-cadherin for selective cell-cell adhesion, P-cadherin is normally undetectable in these pluripotent cells (Hagiwara-Chatani *et al.*, 2017). The homophilic interaction between the characteristic extracellular domain in each cadherin determines the cell-clustering specificity; however, all classical cadherins share binding partners for linkage to the cytoskeletal actin network (Green *et al.*, 2020; Kang *et al.*, 2017). Thus, E-cadherin-expressing cells often exhibit weakened intercellular adhesive properties if they simultaneously express another classical cadherin, such as N- or P-cadherin. This is possibly due to competitive binding to the cytoskeleton (Hagiwara-Chatani *et al.*, 2017; Wheelock *et al.*, 2008). On the other hand, cells displaying E- to N/P-cadherin switch often enhance migratory activity and often undergo epithelial-mesenchymal transition (EMT), which might be attributed to the differences in the adhesive strength, turnover, and signals among these cadherins (Lilien *et al.*, 2002; Thiery *et al.*, 2012).

In the present study, we investigated the functional relationship between extracellular syntaxin4 and P-cadherin in 2D- and 3D-ES cells. We identified the P-cadherin protein as a direct cue for autochthonous and uncontrollable differentiation in ES cells and found that extracellular syntaxin4 triggered upregulation of *P-cadherin*, but its translation was prevented in 3D-ES cells.

## Materials and Methods

### Cell culture and reagents

Naïve mouse ES cells (E14-Tg2A) and their derivatives (those with transgenes) were maintained on gelatin-coated dishes in Glasgow’s Minimum Essential Medium (Fuji Film-Wako, Osaka, Japan) supplemented with 10% fetal bovine serum (FBS), 1 mM glutamine (Thermo Fisher, Tokyo, Japan), 1 mM sodium pyruvate (Fuji Film-Wako), 0.1 mM non-essential amino acids (Sigma-Aldrich, Tokyo, Japan), and 0.1 mM β-mercaptoethanol (Fuji Film-Wako), along with leukemia inhibitory factor (LIF) and MEK/GSK3 inhibitors (2i) (1 μM PD0325901 and 3 μM CHIR99021) (Selleck, Tokyo, Japan). Prior to each experiment, ES cells were precultured for 24 h in the presence of LIF, but without 2i. To prepare EB-like cell aggregates, 750,000 cells were suspended in a 1.5-ml medium containing 1 kU deoxyribonuclease (Sigma-Aldrich), seeded in each well of agarose-coated 6 well-plates, and rotated at 30 rpm. Mouse embryonic teratocarcinoma F9 cells (CRL-1720; ATCC, USA) maintained in Dulbecco’s modified Eagle’s medium/nutrient mixture F12 (Fuji Film-Wako) were used to confirm the effect of extracellular syntaxin4. The proteasome inhibitor lactacystin and lysosomal inhibitor chloroquine were purchased from Sigma-Aldrich and used at concentration of 20 μM and 100 μM, respectively.

### Expression constructs and transfection

The doxycycline (dox)-inducible expression construct for extracellular syntaxin4 has been reported previously (Hagiwara-Chatani *et al.*, 2017). In brief, cDNA of syntaxin4 was fused with those encoding IL-2 signal peptide for extracellular presentation and T7 peptide for detection at the N-terminus. Then the cDNA was subcloned into piggyBac (PB)-based dox-regulatable expression plasmid (Woltjen *et al.*, 2009), which has been additionally introduced with a neomycin-resistance cassette (PBtet0606). To generate expression constructs for P-cadherin, P-cadherin-Venus, or P-cadherin-P2A-Venus, cDNAs encoding these proteins were generated by PCR. Each cDNA was then subcloned downstream of the tetracycline/dox-responsible element in PBtet0606 vector. To establish stable ES cells with inducible expression of the transgene, cells were transfected with a mixture of the generated plasmid, pCAG-PBase, and PB-CA-rtTA-Adv (Addgene, Watertown, USA), the latter of which was additionally introduced with an expression cassette for the hygromycin-resistant gene. Transfection was performed using Lipofectamine 2000 (Invitrogen, Carlsbad, USA) for P-cadherin, or electroporation (Nepa Gene, Ichikawa, Japan) for P-cadherin-Venus and P-cadherin-P2A-Venus. After selection with G418 and hygromycin, the cells were tested for the expression of the transgene in the presence and absence (5 μg/ml) of dox. The tet/dox inducible system has been widely used for functional analyses of several genes in ES cells (Ting *et al.*, 2005), and our previous RNAseq data indicated that dox–treatment gave no significant effects on the expression of mRNAs of syntaxin4 and P-cadherin in parental ES cells (Hagiwara-Chatani *et al.*, 2017). Predictably, we confirmed this is also the case at the protein level (Supplementary Fig. S1A).

### Immunodetection

Immunocytochemistry and immunoblot analyses were performed according to standard procedures with slight modifications. For immunocytochemistry, cells cultured on gelatin-coated glass-bottom dishes (Matsunami, Osaka, Japan) or collagen1A-coated 4-well chamber slides (SPL, Miramar, USA) were used. The samples were counterstained with 4,6-diamidino-2-phenylindole (DAPI; Sigma-Aldrich) and analyzed using a TCS SPE system (Leica, Tokyo Japan). For immunoblot analyses, the signal intensity of the protein band relative to that of β-actin was quantified using Image J software (Schneider *et al.*, 2012). The primary antibodies used in this study included those against the T7-tag (MBL, Tokyo, Japan), P- and E-cadherin (gifts from Dr. Takeichi, Riken Center for Developmental Biology), β-actin (Sigma-Aldrich), Nanog (Abcam, Cambridge, UK), and HIF1-α (a gift from Dr. Imaoka, Kwansei Gakuin University). Horseradish peroxidase, tetramethyl rhodamine, and Alexa488-labeled secondary antibodies were purchased from Sigma-Aldrich or Invitrogen.

### Quantitative reverse transcription PCR (qRT-PCR)

Total RNA was extracted using the RNA Extraction Miniprep System (VIOGENE, Taipei Taiwan), and reverse-transcribed using an RNA-PCR kit (TaKaRa Bio, Kusatsu, Japan). qRT-PCR was performed using Fast Start Essential DNA Green Master on a Right Cycler Nano system (Roche, Basel, Switzerland). The primer pairs used for amplification of *P-cadherin*, *Nanog*, *Oct3/4*, and *β-actin* are listed in Supplementary Table S1. The expression of each mRNA was normalized to that of *β-actin*. In parental ES cells, the mRNA of P-cadherin or that of a stemness marker Oct3/4 was not changed in 3D cell aggregates (Supplementary Fig. S1B).

### Statistical analyses

Data are presented as mean ± standard deviation from at least three independent experiments. The *p*-values were determined by Student’s t-test, with *p*-values <0.05 considered statistically significant. One-way ANOVA and Tukey’s test were performed for multiple comparisons.

## Results and Discussion

### Extracellular syntaxin4 leads to the expression of P-cadherin protein only in 2D-ES cells

**Fig. 1:**
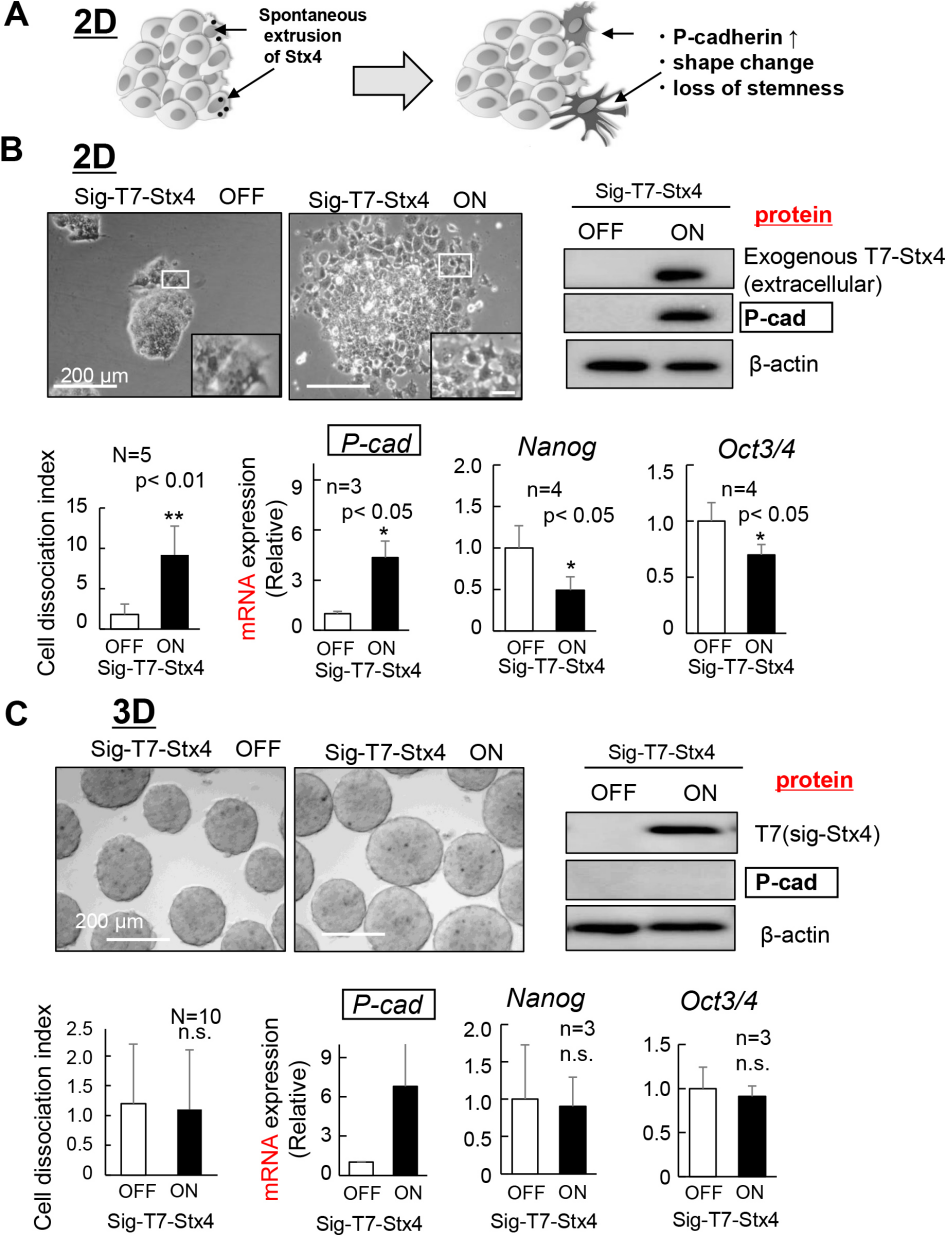
Effects of extracellularly extruded syntaxin4 on the expression of P-cadherin and stemness markers A, Schematic image of our previous results (Hagiwara *et al.*, 2013; Hagiwara-Chatani *et al.*, 2017). ES cells in 2D spontaneously extrude subpopulation of syntaxin4 in the absence of 2i, which leads to upregulation of P-cadherin and loss of stemness. B, In two-dimensional culture, an inducible expression of signal peptide-connected syntaxin4 for 3 days induces flattened morphology and dissociation of cells (upper and lower left), as well as upregulation of P-cadherin and downregulation of stemness markers *Nanog* and *Oct3/4* in ES cells (upper right and lower). Insets, enlarged images. Bars denote 200 μm. Cell adhesive property was quantified by counting the average number of cells dissociated from a colony (2D) or aggregate (3D, see Fig. 1C), and shown as cell dissociation index (lower left). For the immunoblot analyses, the signal intensity of T7-tagged syntaxin4 or P-cadherin, relative to that of β-actin, was quantified using Image J software. C, In 3D, the inducible expression of signal peptide-connected syntaxin4 for three days did not affect the cell adhesive property and expression of the stemness markers (upper and lower left), in which P-cadherin mRNA (*P-cad*), but not the P-cadherin protein, was upregulated. n.s., not significant.

Previously, we showed that mouse ES cells spontaneously extrude syntaxin4 at the cell surface and these cells display enhanced migratory capacity, exit from naïve pluripotency, and upregulate P-cadherin, even in the presence of LIF (Fig. 1A) (Hagiwara-Chatani *et al.*, 2017). Using 2D-ES cells with a dox-inducible expression cassette for signal peptide fused T7-syntaxin4, we clearly confirmed these effects (Fig. 1B and Supplementary Fig. S1C). Although the removal of LIF resulted in a dramatic acceleration of the syntaxin4-induced phenotypic changes (Supplementary Fig. S1D), we decided to use LIF-containing medium for all the experiments, in which cells without expression of the transgene practically maintain the undifferentiated state. We then investigated whether this was also the case in 3D culture, which is supposed to provide a proper environment for subsequent directional differentiation in ES cells. While 2D cells displayed markedly upregulated P-cadherin protein with disruption of intercellular adhesion and downregulation of *Nanog* and *Oct 3/4*, cells in 3D aggregates appeared to retain firm cell-cell adhesion with sustained expression of stemness markers, at least within a few days (Fig. 1B and C). Intriguingly, P-cadherin mRNA was also upregulated in 3D-ES cells, however, P-cadherin protein remained undetectable under these conditions, indicating the posttranscriptional regulation of P-cadherin in 3D-ES cells (Fig. 1C).

### Posttranscriptional suppression of P-cadherin expression in 3D-ES cells

**Fig. 2:**
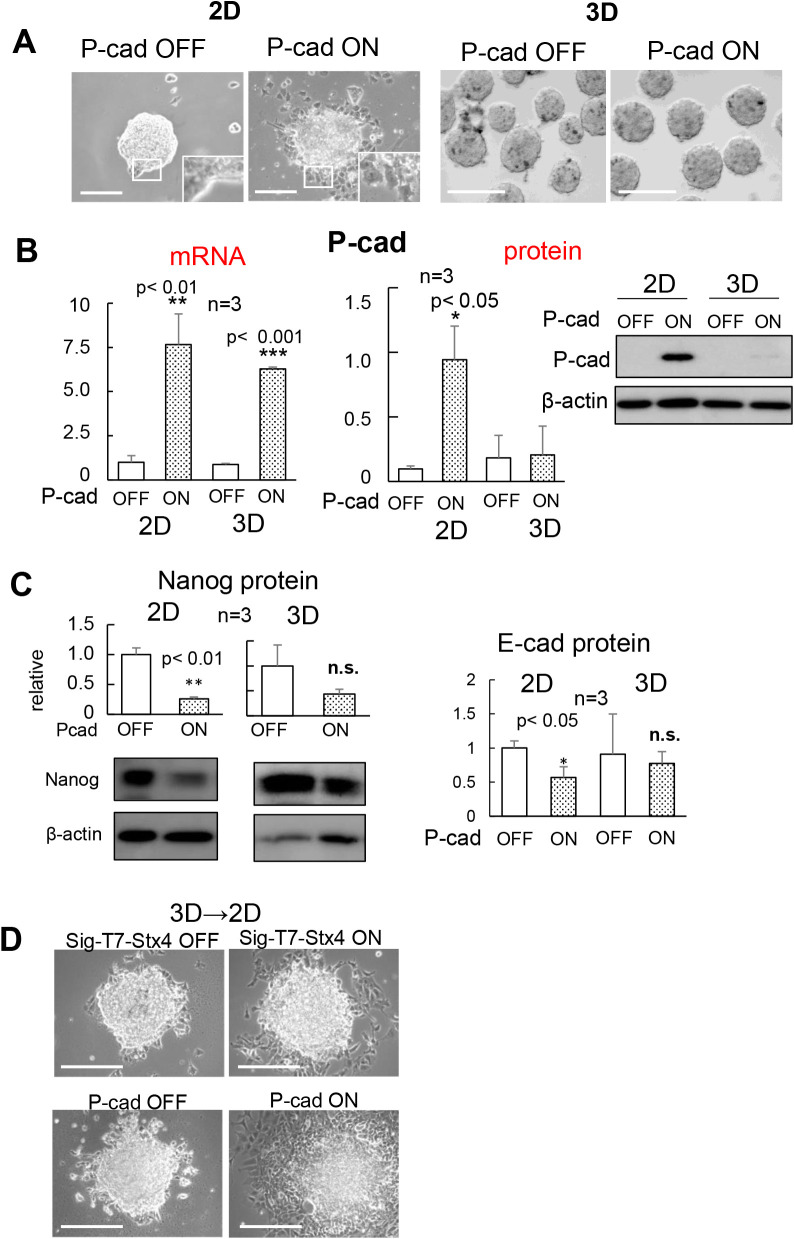
Expression regulation of exogenously introduced P-cadherin in 2D and 3D could account for the effect of extracellular syntaxin4 A, An inducible expression of exogenous *P-cadherin* for three days led to flattened cell morphology with active scattering in 2D, but not in 3D. Insets, enlarged images. Bars, 250 μm. B, Expression of mRNA and protein of P-cadherin (upper), and a stemness marker Nanog (lower) in 2D and 3D. P-cadherin mRNA was effectively produced from the P-cadherin construct, but the expression of P-cadherin protein was suppressed in 3D (upper). C, A significant loss of stemness and a decline in the expression of E-cadherin were observed only in 2D (lower). n.s., not significant. D, Appearance of 3D-ES aggregates on culture dish for 3 days, which allows peripheral cells to migrate in two dimensions. The cell migration and scattering were clearly accelerated in response to inducible expressions either of *sig-T7-stx4* or *P-cadherin*. Bars, 250 μm.

**Fig. 3:**
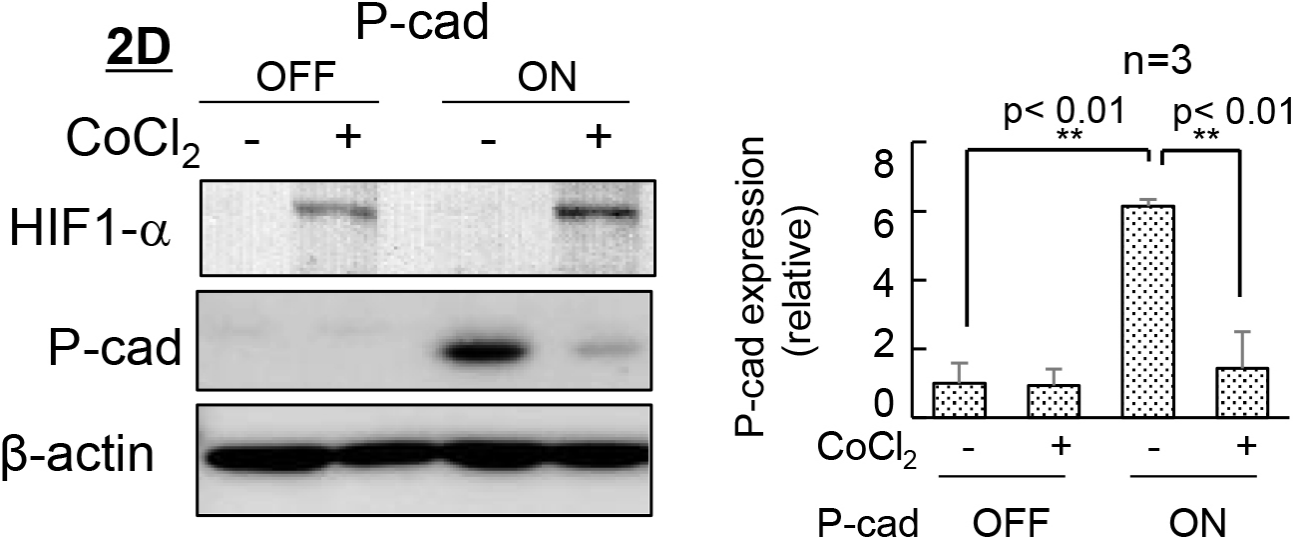
Suppression of expression of P-cadherin protein is attributed to hypoxia A, ES cells in 2D induced expression of HIF-1α with a dramatic suppression of P-cadherin protein upon treatment with a hypoxia-mimetic agent CoCl_2_. Left, immunoblotting; right, quantification of P-cadherin protein.

Given the close relationship between the expression of the P-cadherin protein and changes in cell behavior, we next investigated the effects of the inducible expression of P-cadherin. The induction of P-cadherin transgene expression practically reproduced the effect of extracellular syntaxin4. Obvious expression of P-cadherin protein, alterations in cell shape/mobility, and decreased stemness were induced in 2D, but not in 3D (Fig. 2A and B). In addition, the expression of P-cadherin protein concurrently induced downregulation of E-cadherin (Fig. 2C), as has been induced by extracellular syntaxin4 (Hagiwara-Chatani *et al.*, 2017). When 3D-ES cells, either with the expression construct of extracellular syntaxin4 or P-cadherin, were transferred from the agarose-coated dish to the culture dish to allow peripheral cells to attach and subsequently migrate in two-dimensions, these cells rapidly migrated in response to extracellular syntaxin4 and P-cadherin (Fig. 2D and Supplementary Fig. S2). These results suggest that P-cadherin is a critical cue for autochthonous differentiation in ES cells, and that this protein mediates, at least in part, the effects of spontaneously extruded syntaxin4. While P-cadherin has been identified as a cell-cell adhesion molecule, several reports have revealed its additional functions, such as activation of receptor tyrosine kinases, induction of tumor metastasis, and upregulation of EMT transcription factors, especially in cells co-expressing E-cadherin (Paredes *et al.*, 2007; Vieira and Paredes, 2015). Given that the expression of P-cadherin protein resulted in the downregulation of E-cadherin protein, and that classical cadherins have a conserved cytoplasmic domain and share elements for linkage to the actin cytoskeleton (Green *et al.*, 2020; Kang *et al.*, 2017), P-cadherin might weaken intercellular adhesion mediated by E-cadherin. Indeed, syntaxin4-induced changes in cell behavior and P-cadherin expression profile were clearly induced in embryonic carcinoma F9 cells that originally expressed E-cadherin, but not P-cadherin (Supplementary Fig. S3). Next, we investigated the possible mechanism of the suppressed expression of P-cadherin protein in 3D-ES cells. 3D culture reportedly provide cells with severe hypoxic conditions (Wu *et al.*, 2014), in which undifferentiated cells retain undifferentiated states (Di Mattia *et al.*, 2021; Ezashi *et al.*, 2005). In addition, the expression of the P-cadherin protein was shown to be negatively correlated with hypoxia-inducible factor-alpha (HIF1-α) in another cell type (Sousa *et al.*, 2014). To test the possible involvement of hypoxia in the posttranscriptional inhibition of P-cadherins, 2D-ES cells with the P-cadherin transgene were incubated with the hypoxic stress inducer CoCl_2_. This treatment upregulated HIF1-α, and the production of P-cadherin protein was severely prevented, suggesting the causal involvement of hypoxia in the abortive expression of P-cadherin protein in 3D-ES cells (Fig. 3). It is notable, however, the outermost non-hypoxic cell populations in the 3D aggregates were also negative for P-cadherin protein. Given that signaling from focal adhesion is well known to decrease ES cell stemness (Taleahmad *et al.*, 2017), it is reasonable to hypothesize that cell adhesion to the substrate is also required for the expression of P-cadherin protein.

### P-cadherin expression in ES cells might be regulated in the translational process

**Fig. 4:**
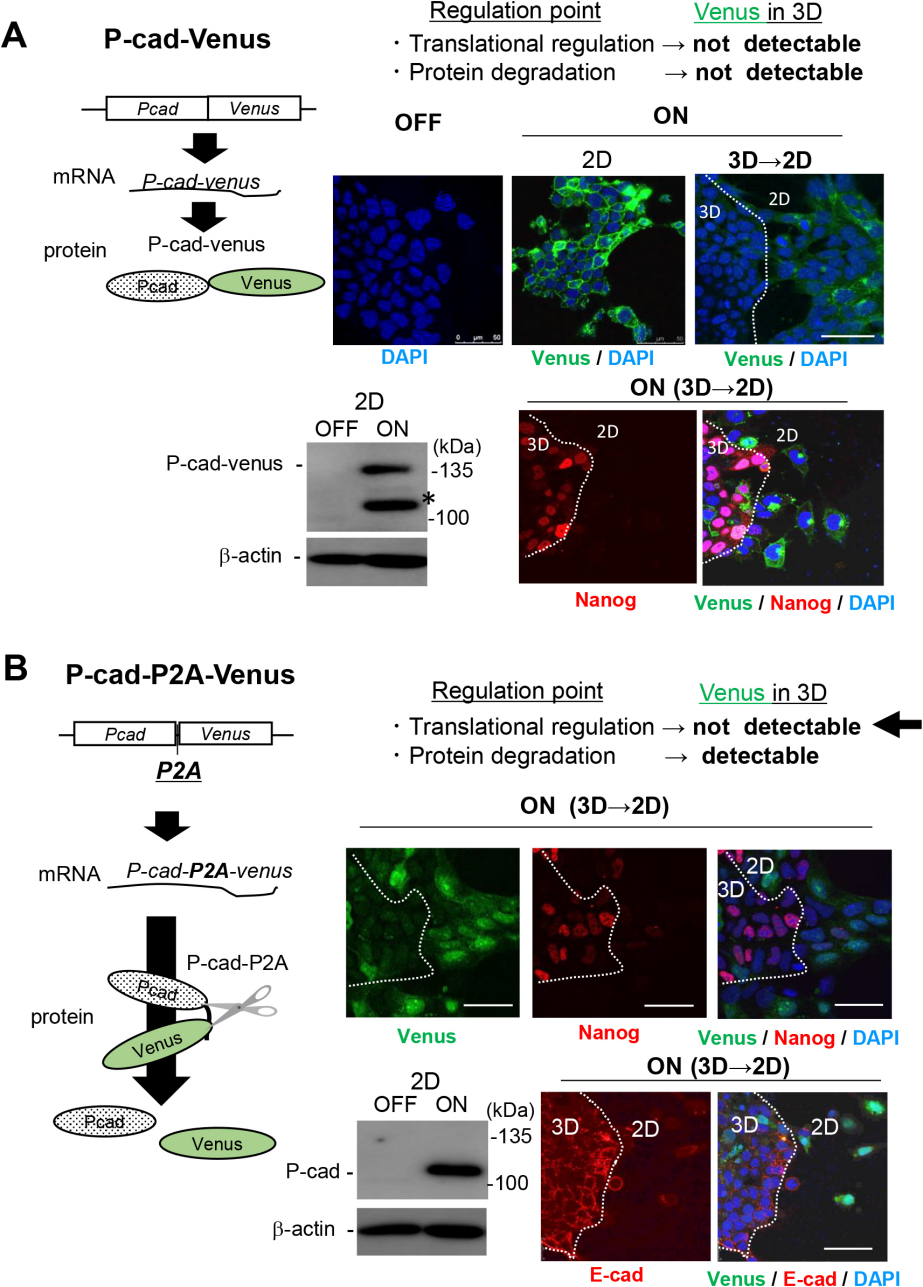
Expression of P-cadherin protein might be regulated at the translation process of P-cadherin mRNA A, Upper left, a schematic image of the production of the transgene product from P-cadherin-Venus expression cassette (a fusion protein). Lower left, the expression of P-cadherin-Venus fusion protein upon Dox-treatment (ON) was detected with anti-P-cadherin monoclonal antibody. An unexpected protein band resembling to Venus-free P-cadherin was also detected (*), which might be generated during sample preparation for immunoblotting, given that this was never detected in control cells (dox -). Upper right, Venus fluorescence (green) was observed at the cell-cell contact site only of 2D-ES cell populations, confirming the suppressed expression of the fusion protein in 3D cells. Lower right, a stemness marker Nanog (red) became almost undetectable in the fusion protein-positive 2D-ES cell populations. Nuclei were counterstained with DAPI (blue). Bars, 50 μm. B, Upper left, a schematic image of the production of the transgene product from P-cadherin-P2A-Venus expression cassette (P-cadherin and Venus protein). Using an expression cassette, the single mRNA of *P-cadherin-P2A-Venus* is transcribed, which is subsequently translated in stroke. The Venus protein exists independent of P-cadherin, because of self-cleave in P2A sequence. Lower left, the intact P-cadherin protein (118 kDa) was detected in 2D cells. Right, the expression of Venus (green in upper panels), P-cadherin (green in lower panels), together with the offset of the stemness marker Nanog (red in upper panels) were detected predominantly in 2D cell populations (upper). In the lower panels, E-cadherin was also visualized (red in lower panel). These staining patterns might exclude the possible involvement of protein elimination systems in the expression regulation of P-cadherin protein.

Finally, we tried to clarify whether the expression of P-cadherin protein was regulated during the translation process or by proteolytic elimination systems in 3D-ES cells. As a premier evaluation assay, ES cells introduced with the P-cadherin-Venus fusion protein were aggregated and seeded onto culture dishes to allow the peripheral cell populations to attach and migrate onto substrates, creating a mixture of 2D and 3D cells. In this culture, Venus fluorescence was observed only in 2D-migrating cells at cell-cell contact sites, confirming the posttranscriptional regulation of P-cadherin expression (Fig. 4A). In addition, 2D-cells expressing the fusion protein lost the stemness marker Nanog (Fig. 4A). The expression profile of P-cadherin protein was not altered, even in the presence of the proteasome inhibitor lactacystin or the lysosomal degradation inhibitor chloroquine (data not shown). These results imply that the suppressed expression of P-cadherin protein in 3D aggregates is caused in the process of mRNA translation. Based on these findings, we next introduced ES cells with an expression cassette for similar but distinct fusion proteins (i.e., in-frame) for the P2A sequence (Liu *et al.*, 2017) inserted between P-cadherin and Venus, in order to confirm the translational regulation for the expression of P-cadherin. From this expression cassette, P-cadherin and Venus are initially translated as a fusion protein, then immediately, disconnected because of the P2A sequence, which results in the independent expression of plasmalemmal P-cadherin and cytoplasmic Venus. Hence, this construct would enable us to judge the suppressed expression of P-cadherin is due to the protein degradation or translational repression by testing whether Venus fluorescence in 3D-Es cells is observed or not. Experimental results showed that Venus fluorescence was detected only in the cytoplasm of 2D-migrated cells, but not in the 3D-cells, suggesting that P-cadherin expression might be regulated during the translation process in 3D-ES cells (Fig. 4B). If this is the case, such mechanism is very unique, since translational suppression is generally instructed by untranslated regions (Jia *et al.*, 2013; Leppek *et al.*, 2018), whereas exogenous *P-cadherin* only share the coding sequence with endogenous *P-cadherin*.

**Fig. 5:**
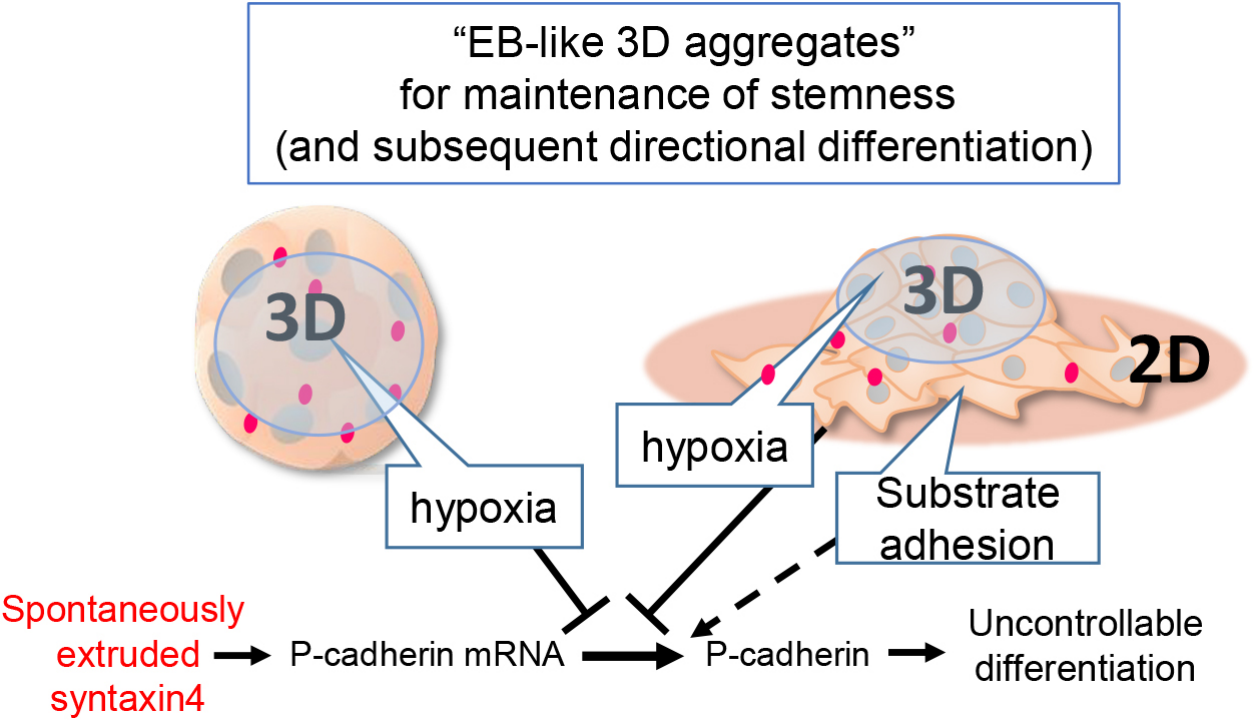
Schematic diagram of stemness maintenance model in EB-like cell aggregates ES cells autochthonously extrude syntaxin4, which leads to upregulation of P-cadherin for the cell shape change and onset of differentiation. The hypoxic and substrate-free conditions created in EB-like 3D-cell aggregates suppress the translation of P-cadherin mRNA provoked by extruded syntaxin4 to prevent spontaneous differentiation.

In conclusion, the cellular microenvironment created in EB-like 3D cell aggregates has been shown to enhance the cellular potency for stemness maintenance and subsequent directional differentiation. (Brickman and Serup, 2017; Karanfil and Bagci-Onder, 2016). That spontaneously extruded syntaxin4 upregulates *P-cadherin* transcription but its translation is hindered in 3D might provide a new mechanistic insight into this phenomenon (Fig. 5).
