# Supplementary material for: Suppression of P-cadherin expression as a key regulatory element for embryonic stem cell stemness
Source: Cell Struct Funct. 2022 Dec 28;48(1):49–57. doi: 10.1247/csf.22060 (PMC10721948; doi:10.1247/csf.22060)
Supplement: Supplementary file 1 — Supplementary Materials [file csf_48_22060_1.pdf]

Takeda Y ＆Matsuguchi S et al., Supplementary Table1

Primer pairs used in this study

P-cadherin	GCACTGCTGACCCTTCTACTG<br>GGGCTCTTTGACCTTCCTCT
Nanog	TTCTTGCTTACAAGGGTCTGC<br>CAGGGCTGCCTTGAAGAG
Oct3/4	GTTGGAGAAGGTGGAACCAA<br>CTCCTTCTGCAGGGCTTTC
β-actin	CCTCACCCTCCCAAAAGC<br>GTGGACTCAGGGCATGGA

Takeda Y ＆Matsuguchi S et al., Supplementary Figure S1

A Parental ES cells

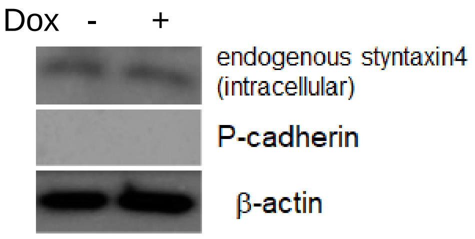

A, Effect of doxycycline treatment (Dox +) for 3 days on the expression of endogenous intracellular syntaxin4 (Stx4) and P-cadherin protein in parental ES cells. β-actin, loading control. Most of endogenous syntaxin4 was shown to be expressed at the cytoplasmic surface of the cell (Hagiwara-Chatani et al. 2017). No obvious effects of Dox were detected.

B Parental ES cells

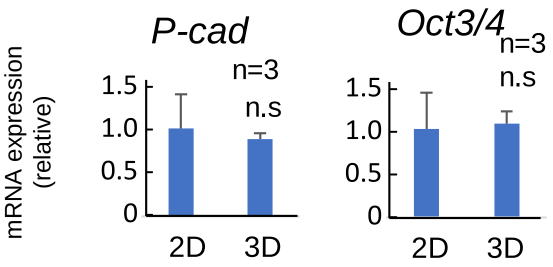

B, Amount of mRNA of P-cadherin (*P-cad*) or Oct3/4 (*Oct3/4*) was not changed if parent ES cells were cultured as 3D-aggregates

C Transfectants (Sig-Stx4)

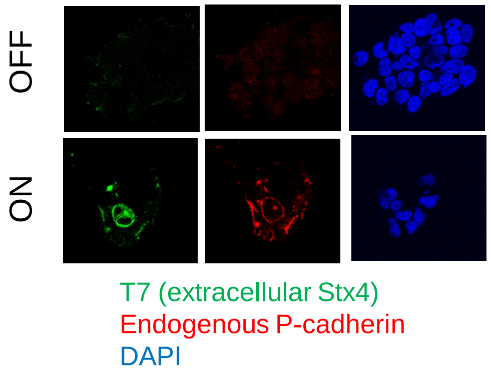

C, Expression of extracellular syntaxin4 induced expression of P-cadherin protein. When extracellular expression of exogenous syntaxin4 (Sig-Stx4) was induced for a few days (ON), endogenous P-cadherin became detectable (red).

D Transfectants

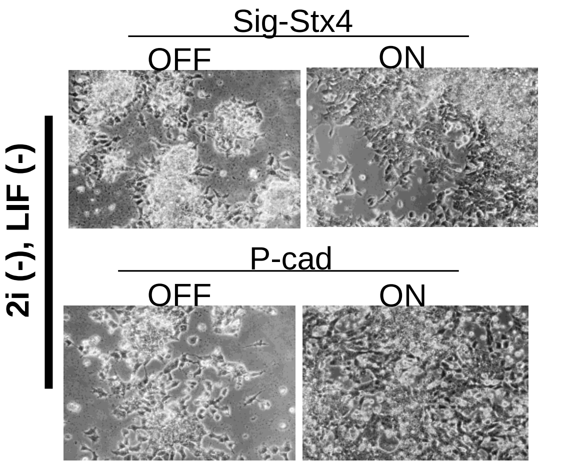

D, Effects of extracellular syntaxin4 (Swig-Stx4) and P-cadherin might be LIF-independent. Induction of these transgenes (ON) in ES cells dramatically accelerated the migration in normal medium (without 2i and LIF).

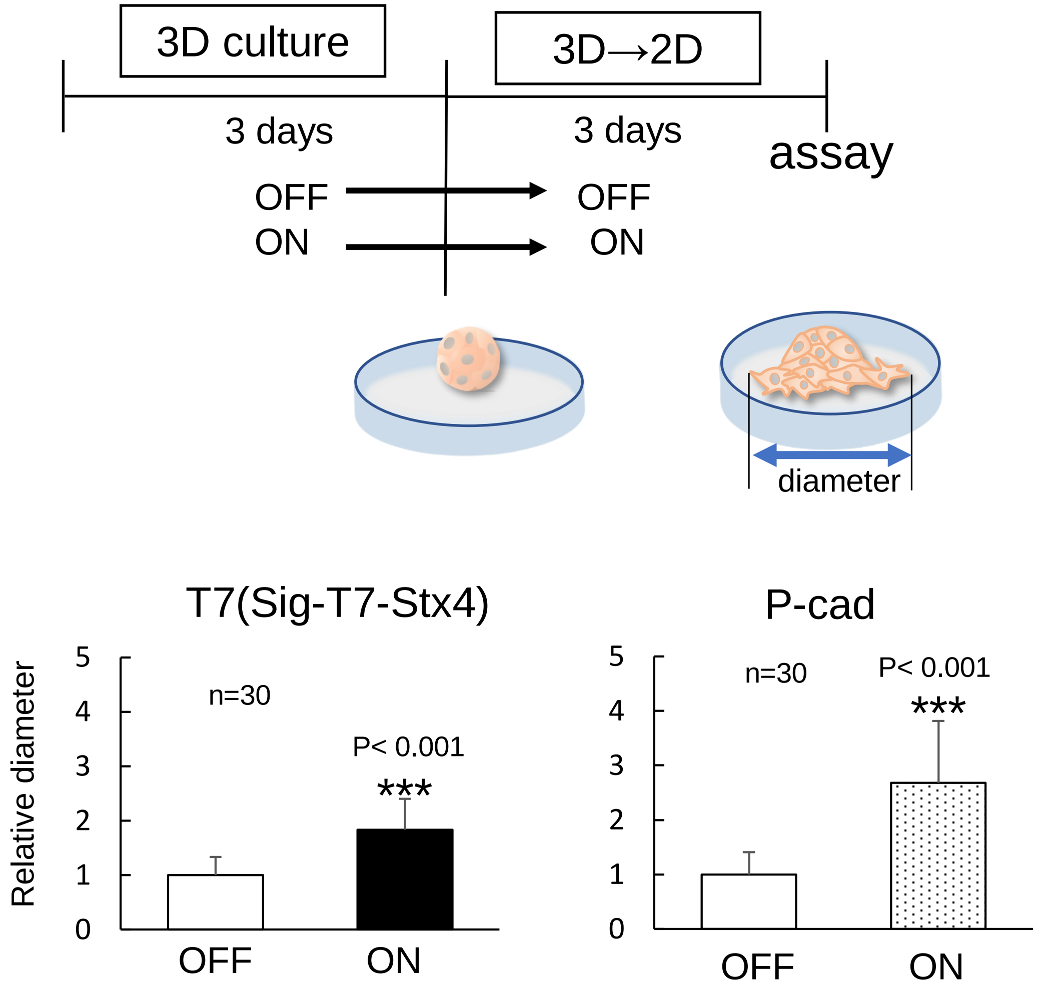

Supplementary Fig. S2. Quantification of 2D cell migration/spreading of 3D-ES cell aggregates placed onto culture dishes for 3 days. Upper, experimental setup for the analysis. Sizes of ES cell aggregates with and without induction of *sig-T7-Stx4* or *P-cad* for 3 days were almost the same as shown in Figure 1c and 2a. These cell aggregates were seeded onto the culture dish and the diameters of the spreading colonies were measured after 3 days of incubation. Active migration and spreading in 2D cell populations were apparent in response to the re-expression of sig-T7-Stx4 or P-cadherin.

Takeda Y & Matsuguchi S et al., Supplementary Figure S3

F9 (embryonic carcinoma) Sig-T7-Stx4

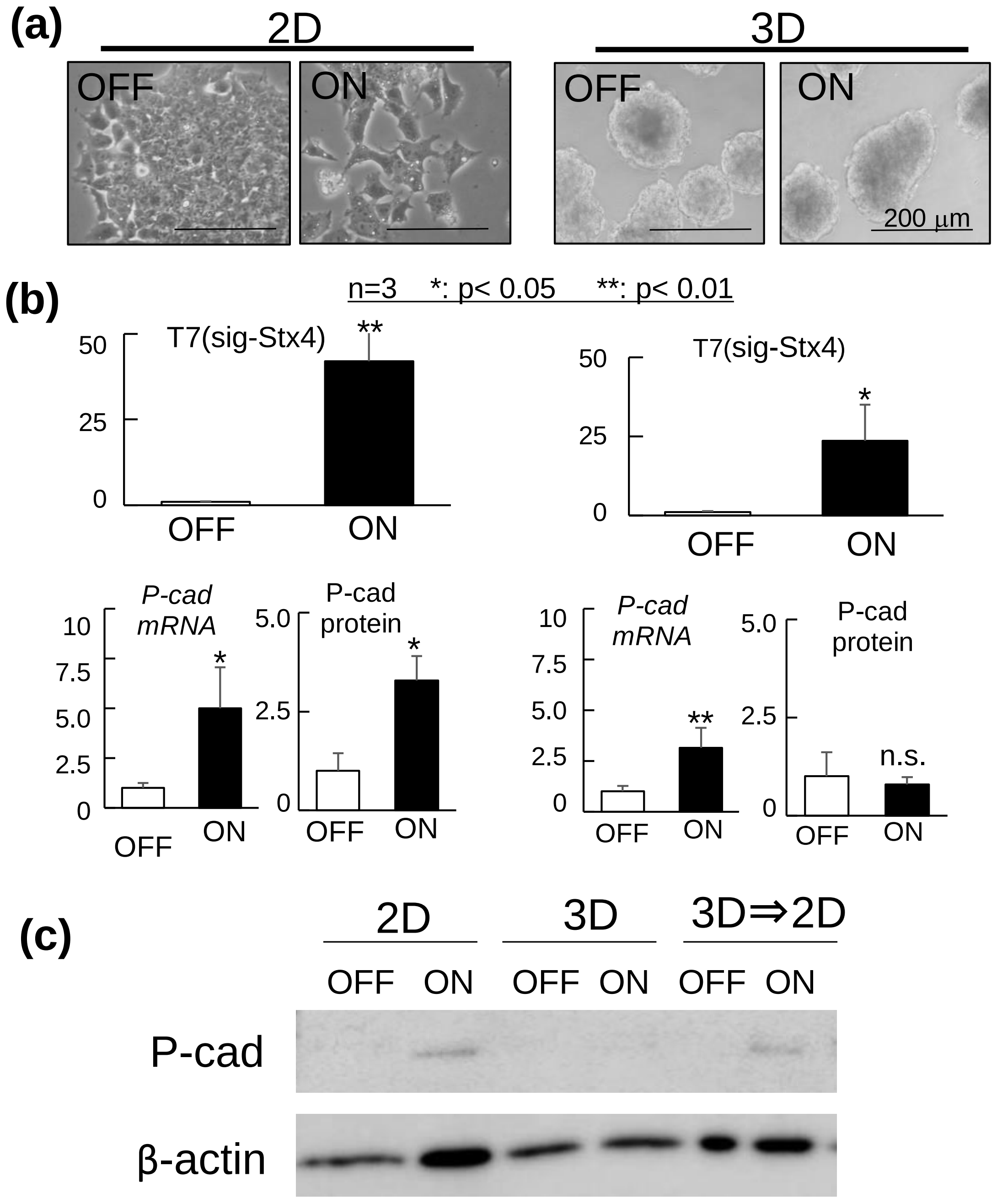

Supplementary Fig. S3. Extracellular syntaxin4-induced upregulation of *P-cadherin* and the 3D-dependent suppression of P-cadherin translation is also the case in embryonic carcinoma F9 cells. (a), Phenotypic appearances of F9 cells with (ON) and without (OFF) induction of extracellular syntaxin4 (Stx4) in 2D and 3D. (b), Expression of extracellular Stx4 (T7(sig-Stx4)), P-cadherin mRNA (*P-cad*), and P-cadherin protein (P-cad protein). Extracellular syntaxin4 upregulates *P-cad*, but the expression of P-cadherin protein was not observed in 3D. (c), When F9 cell aggregates were seeded onto a culture dish to allow 2D migration and spreading, the expression of P-cadherin protein was restored.
